# Therapeutic roles of natural remedies in combating hereditary ataxia: A systematic review

**DOI:** 10.1186/s13020-020-00414-x

**Published:** 2021-01-28

**Authors:** Michael Weng Lok Phang, Sze Yuen Lew, Ivy Chung, William Kiong-Seng Lim, Lee Wei Lim, Kah Hui Wong

**Affiliations:** 1 Department of Anatomy, Faculty of Medicine, University of Malaya, Kuala Lumpur, 50603 Malaysia; 2 Department of Pharmacology, Faculty of Medicine, University of Malaya, Kuala Lumpur, 50603 Malaysia; 3 Faculty of Medicine and Health Sciences, Universiti Malaysia Sarawak, Kuching, Sarawak, 94300 Malaysia; 4 Neuromodulation Laboratory, School of Biomedical Sciences, Li Ka Shing Faculty of Medicine, The University of Hong Kong, 21 Sassoon Road, Pokfulam, Hong Kong Special Administrative Region China

**Keywords:** Hereditary ataxia, Natural remedy, Medicinal plant, Herbal formulation, Medicinal mushroom

## Abstract

**Background:**

Hereditary ataxia (HA) represents a group of genetically heterogeneous neurodegenerative diseases caused by dysfunction of the cerebellum or disruption of the connection between the cerebellum and other areas of the central nervous system. Phenotypic manifestation of HA includes unsteadiness of stance and gait, dysarthria, nystagmus, dysmetria and complaints of clumsiness. There are no specific treatments for HA. Management strategies provide supportive treatment to reduce symptoms.

**Objectives:**

This systematic review aimed to identify, evaluate and summarise the published literature on the therapeutic roles of natural remedies in the treatment of HA to provide evidence for clinical practice.

**Methods:**

A systematic literature search was conducted using the Preferred Reporting Items for Systematic Reviews and Meta-Analyses (PRISMA). Web of Science, PubMed and Science Direct Scopus were thoroughly searched for relevant published articles from June 2007 to July 2020.

**Results:**

Ten pre-clinical and two clinical studies were eligible for inclusion in this systematic review. We identified the therapeutic roles of medicinal plants *Brassica napus, Gardenia jasminoides, Gastrodia elata, Ginkgo biloba, Glycyrrhiza inflata, Paeonia lactiflora, Pueraria lobata* and *Rehmannia glutinosa*; herbal formulations Shaoyao Gancao Tang and Zhengan Xifeng Tang; and medicinal mushroom *Hericium erinaceus* in the treatment of HA. In this review, we evaluated the mode of actions contributing to their therapeutic effects, including activation of the ubiquitin–proteasome system, activation of antioxidant pathways, maintenance of intracellular calcium homeostasis and regulation of chaperones. We also briefly highlighted the integral cellular signalling pathways responsible for orchestrating the mode of actions.

**Conclusion:**

We reviewed the therapeutic roles of natural remedies in improving or halting the progression of HA, which warrant further study for applications into clinical practice.

## Background

Ataxia is defined as the loss of full control of bodily movements, and can be inherited, acquired, or occur sporadically [1]. Hereditary ataxia (HA) represents a group of genetically heterogeneous neurodegenerative diseases resulting from dysfunction of the cerebellum or disruption of connection between the cerebellum and other areas of the central nervous system [2]. Hereditary ataxia is characterised by pyramidal syndrome, movement disorder, seizure, peripheral neuropathy and cognitive dysfunction [3]. Furthermore, HA patients often present with unsteadiness of stance and gait, dysarthria, nystagmus, dysmetria and clumsiness [4]. Classification of the subtypes of HA is very challenging due to the many neurological and metabolic diseases associated with cerebellar dysfunction, and the phenotypic heterogeneity among the hereditary disorders [5]. Nevertheless, HA can be classified by the pattern of inheritance: autosomal dominant, autosomal recessive and X-linked ataxia [3].

Among the autosomal dominant cerebellar ataxias (ADCA), spinocerebellar ataxia (SCA) 3 is the most common subtype, followed by SCA1, 2, 6, 7 and 17, which are all caused by the expansion of polyglutamine-coding Cytosine-Adenine-Guanine (CAG) repeats [6]. Another ACDA subtype is episodic ataxia (EA), which include the common EA1 and 2 subtypes [2]. Among the autosomal recessive cerebellar ataxias (ARCA), Friedreich’s ataxia (FRDA) is the most common subtype, followed by ataxia-telangiectasia (AT), and ataxia oculomotor apraxia type 1 (AOA1) and type 2 (AOA2) [7]. The most common X-linked ataxia is fragile X-associated tremor ataxia syndrome (FXTAS). The age of onset varies according to types of HA, but onset actually helps in the diagnosis of HA: infancy onset suggests AT; childhood or teenage onsets suggest EA, FRDA, AOA1 and AOA2; and adult onset suggests SCA, FXTAS, or other autosomal recessive cerebellar ataxias [5]. Table 1 summarises the highly prevalent types of HA and their specific ethnic distributions, gene and locus of mutation, and clinical presentations.

**Table 1:** Common forms of hereditary ataxia (HA)

Hereditary ataxia	Ethnicity	Gene and locus of mutation	Clinical presentation		Neuroimaging finding	Neurometabolite finding	Biochemical finding	Reference
			Motor symptom	Non-motor symptom				
Autosomal dominant								
SCA1	Russian, Polish, Indian, Japanese and Eastern Siberian	ATXN1 on chromosome 6p22.3	Gait ataxia, pyramidal, extrapyramidal and bulbar signs, dysarthria, nystagmus, chorea and peripheral neuropathy	Cognitive impairment and respiratory failure	Atrophy of the cerebellum, brainstem and cerebral frontal lobe	Decreased glutamate, tNAA, NAA/Cr ratio and Cho/Cr ratio Increased glutamine, myoinositol and tCr	Increased lactate	[6–12]
SCA2	Cuban, Korean, South African and Indian	ATXN2 on chromosome 12q24.12	Gait ataxia, pyramidal and extrapyramidal signs, dysarthria and dysphagia	Cognitive impairment, psychiatric symptoms, sleep disturbances and slow saccadic eye movements	Atrophy of cerebellum, brainstem, cerebral frontal lobe, spinal cord and cranial nerves III to XII	Decreased glutamate, tNAA, NAA/Cr ratio and Cho/Cr ratio Increased myoinositol and tCr	Decreased GSH, GST, SOD, CAT, zinc, iron, copper, erythropoietin, dopamine metabolites and ethanolamine Increased MDA	[6, 8–14]
SCA3/MJD	Portuguese, Japanese, Taiwanese, German and Brazilian	ATXN3 on chromosome 14q32.12	Gait ataxia, pyramidal and extrapyramidal signs, nystagmus, lid retraction, decreased saccade velocity, amyotrophy fasciculations and peripheral neuropathy	Mild cognitive impairment, sleep disturbances and involuntary weight loss	Atrophy of cerebellum, brainstem, cerebellar peduncle and cranial nerves III to XII	Decreased glutamate, tNAA, NAA/Cr ratio, Cho/Cr ratio, tNAA/tCr ratio and Glx/tCr ratio Increased myoinositol and tCr	Decreased GPx, l-Proline and l-Tryptophan Increased palmitoleic acid (FFA 16:1) and linolenic acid (FFA 18:3)	[6, 7, 9–12, 15–17]
SCA6	Japanese, American, German, Australian and Dutch	CACNA1A on chromosome 19p13.13	Gait ataxia, pyramidal signs, dysarthria, nystagmus, intention tremor, dystonia and dysphagia	Diplopia	Atrophy of cerebellum	Decreased NAA and NAA/Cr ratio Increased myoinositol	Increased glucose, taurine and lactate	[6, 8, 10–12]
SCA7	South African, Mexican and Scandinavian	ATXN7 on chromosome 3p14.1	Gait ataxia, pyramidal signs, slow eye movements, dysmetria, hyperreflexia, dysarthria, dysphagia and ophthalmoplegia	Visual loss with retinopathy, cognitive impairment and psychiatric symptoms	Atrophy of cerebellum, brainstem, cerebral peduncle and spinal cord	Decreased glutamate and tNAA Increased myoinositol and tCr	Increased lipid hydroperoxides, MDA, protein carbonyls, GR, GPx and paraoxonase	[6, 9–11, 18, 19]
SCA17/HDL4	Japanese, German and Italian	TBP on chromosome 6q27	Gait ataxia, pyramidal and extrapyramidal signs, chorea and myoclonus	Cognitive impairment and psychiatric symptoms	Atrophy in cerebellum, brainstem and cerebrum	N/A	Decreased presynaptic dopamine transporters	[7, 12, 20, 21]
EA1	Australian, Caucasian, American and Russian	KCNA1 on chromosome 12p13	Gait ataxia (attack last from seconds to minutes), dysarthria, seizure, myokymia, neuromyotonic and tremor	Vertigo, difficulty in breathing and diaphoresis	Normal brain	N/A	Increased GABA release	[7, 22–24]
EA2	Caucasian and Korean	CACNA1A on chromosome 19p13	Gait ataxia (attack last from minutes to hours), dysarthria, seizure, nystagmus and dystonia	Vertigo, nausea, headache, diplopia, tonic upward gaze and cognitive impairment	Atrophy of cerebellum	Decreased Cr and high-energy phosphate ratio	Increased lactate	[7, 11, 22, 23, 25, 26]
Autosomal recessive								
FRDA	Caucasian, Middle East and North African	FXN on chromosome 9q21.11	Gait ataxia, eye movement abnormalities, dysarthria, dysphagia, lower extremities weakness and peripheral neuropathy	Skeletal deformities, hypertrophic cardiomyopathy, diabetes, deafness and optic atrophy	Atrophy of cerebellum and cervical spinal cord	Decreased NAA/Cr ratio and Cho Increased glutamine, myoinositol and tCr	N/A	[7, 11, 27, 28]
AT	Iberian, Polish and Russian	ATM on chromosome 11q22.3	Gait ataxia, extrapyramidal signs, oculomotor apraxia, hypotonia, areflexia and peripheral neuropathy	Oculocutaneous telangiectasia, immunodeficiency, cancer susceptibility, sterility, extreme radio sensitivity, diabetes and pulmonary diseases	Atrophy of cerebellum and brainstem	Decreased NAA, Cho, NAA/Cho ratio and Cr Increased Cho and Cho/Cr ratio	Decreased IgA, IgM, IgG2, IgE and absence of ATM protein Increased AFP	[1, 7, 11, 29–31]
AOA1	Japanese and Portuguese	APTX on chromosome 9p21.1	Gait ataxia, extrapyramidal signs, oculomotor apraxia, areflexia, dysarthria, choreoathetosis, oculomotor apraxia and peripheral neuropathy	Cognitive impairment and skeletal deformities	Atrophy of cerebellum	N/A	Decreased albumin level Increased creatine kinase and total cholesterol	[7, 32, 33]
AOA2	Eastern French	SETX on chromosome 9p34.13	Gait ataxia, pyramidal and extrapyramidal signs, oculomotor apraxia, chorea, upper motor neuron signs, head tremor, strabismus and peripheral neuropathy	Mild cognitive impairment	Atrophy of cerebellum	Decreased glutamate, NAA and NAA/Cho ratio Increased myoinositol	Increased creatinine kinase, total cholesterol, AFP, IgG and IgA	[7, 11, 32, 34]
X-linked								
FXTAS	Caucasian	FMR1 on X chromosome	Gait ataxia, intention tremor, eye movement abnormalities and peripheral neuropathy	Cognitive impairment, psychiatric symptoms, hearing loss and dysautonomia	Atrophy of cerebellum and cerebrum	Decreased NAA	N/A	[35–37]

*AFP* alpha fetoprotein, *AOA* ataxia oculomotor apraxia, *AT* ataxia-telangiectasia, *Cho* choline compounds, *Cr* creatine, *EA* episodic ataxia, *FRDA* Friedreich’s ataxia, *FXTAS* fragile X tremor-ataxia syndrome, *GABA* γ-aminobutyric acid, *Glx* glutamate + glutamine, *GST* glutathione S-transferase, *GPx* glutathione peroxidase, *GR* glutathione reductase, *GSH* glutathione, *HDL4* Huntington disease-like 4, *Ig* immunoglobulin, *MDA* malondialdehyde, *MJD* Machado-Joseph disease, *N/A* not applicable, *NAA N*-acetylaspartate, *NAAG N*-acetylaspartatylglutamate, *PCr* phosphocreatine, *SOD* superoxide distmutase, *SCA* spinocerebellar ataxia, *tCr* total creatine (Cr + PCr), *tNAA* total NAA (NAA + NAAG)

Generally, HA is first diagnosed by assessing the history of the presenting condition, past medical history, and personal history, and then by neurological evaluation, systemic physical evaluation, and pertinent paraclinical tests. Laboratory tests can rule out acquired ataxia and identify biomarkers specific to the HA. Neuroimaging using magnetic resonance imaging (MRI) and computed tomography (CT) are useful to determine the presence of cerebellar atrophy or anomalies. After excluding acquired ataxia, the mode of inheritance is then evaluated. In this context, the age of onset and clinical manifestation can well-describe the HA and may provide crucial clue(s) to identify the mutated gene. Molecular genetic testing for HA with high prevalence such as SCA and FRDA is usually suggested before pursuing other extensive tests [21]. If single gene testing does not provide a molecular diagnosis, then high-throughput next-generation sequencing (NGS) such as multigene panel testing, whole-exome or whole-genome sequencing can be conducted to identify the mutated genes. Specialised genetic counselling and risk assessment can then be provided once the pathogenic mutation has been identified [5].

To date, there are no effective treatments for HA and current therapeutic strategies only focus on symptomatic and supportive management [38]. The complexity in the neurochemistry of HA suggests that there may be multiple therapeutic targets. For example, some pharmacological molecules have been used to target motor and non-motor symptoms associated with HA [39]: riluzole, amantadine and varenicline were demonstrated to reduce the deteriorating symptoms in various ADCA and ARCA [4]; acetazolamide and 4-aminopyridine (4-AP) were reported to be effective in reducing symptoms in EA; and acetyl-DL-leucine was found to improve gait variability in patients with various subtypes of cerebellar ataxia including SCA [40]. N-acetyl-L-leucine is currently being tested in a multinational clinical trial as a treatment for AT [41]. Nevertheless, many pharmacological molecules have yet to reach clinical trials or be translated into breakthrough treatments, mainly due to the limited pre-clinical studies [6]. More efforts are needed to discover safe therapeutic agents for HA [42].

Natural remedies have been used as complementary therapeutic agents for various neurodegenerative diseases [43, 44]. Natural remedies are a good source for the discovery of drugs or agents that have therapeutic potential. In addition, holistic practitioners commonly use natural remedies, as they are well accepted and relatively safe compared to synthetic drugs. One of the key distinguishing attributes of natural remedies is their affinity for the target protein or specific biomolecule in living organisms [44]. The distinct features of biological compounds including more chiral centres; more carbon, hydrogen and oxygen and less nitrogen; higher molecular weight and higher polarities compared to synthetic compounds [45]. Biological compounds also contain more sp^3^-hybrid carbon atoms allowing the tetrahedron carbons to form flexible chains or cyclic structures, whereas the multi-functional groups make them bind to the biological targets strongly and increase the additional interactions with biological molecules [46]. Natural metabolites with greater structural diversity, bioactivity and complexity are capable of becoming the substrate for one or more of the transporter systems and to be delivered to the targeted intracellular site of action [47].

Besides, with the recent shift in the mode of discovery from “one-target, one-drug” to “network-target, multiple-component-therapeutics”, many natural remedies or compounds with broad safety profiles have great potential as they have proven efficacy on multiple targets in different diseases [48]. The concept of multiple-target compounds involves the merging of active substructures or the fusion of two or more biological active moieties into one compound in the enhancement of biological activities [49]. Curcumin, resveratrol, quercetin, green tea catechins and salicylate are some of the well-studied biological compounds with multiple molecular targets and mechanism of action elucidated [50]. However, a disadvantage of biological compounds with multiple targets is the ability to bind to multiple proteins of unrelated structures or the unintended off-targets, which may result in potential toxicity and adverse effects [51]. Therefore, it is crucial to characterise and optimise the structure of biological compounds to boost their affinity against the disease-related targets and weaken the activity towards undesired adverse targets [50].

Chinese medicines or traditional Chinese medicines (TCM) have a long history of use in Chinese communities in East and Southeast Asia since ancient times. The basic concepts of TCM originated from the ancient naturalistic philosophies namely *qi*, *yin yang* and the five phases (*wu xing*) theory included wood (*mu*), fire (*huo*), earth (*tu*), metal (*jin*) and water (*shui*) [52]. TCM is a holistic approach as it focuses on the integrity of the human body and its close relationship with social and natural environment. Numerous natural therapeutic methods such as mind-spiritual methods (*qi gong* and *tai chi*) and natural methods (acupuncture, moxibustion and herbal medicine) were applied in TCM to maintain overall health and enhance body’s resistance to disease. The accumulation of empirical experience and collection of observational data in clinical settings are responsible in the development of TCM over the centuries [53].

Demand for TCM continues to grow in the Western world. Application of modern disciplines in TCM has resulted in the isolation and identification of novel and/or active compounds. Some of these were developed to produce new drugs for the treatment of important diseases such as malaria, vascular diseases and cancer which is deemed necessary to improve the quality of TCM materials. However, a majority of TCM has not been elucidated chemically and pharmacologically and most of the TCM-derived compounds were found not to be as active as the original TCM preparation. This points to the possibilities of synergism being involved [48]. Herbal formulation that contains two or more herbs in specific proportions have been used to treat a variety of symptoms in a wide range of diseases, such as forgetfulness, disorientation, insomnia, unconsciousness, cramps and seizures [54].

Therefore, it is a challenging task to develop evidence-based Chinese medicine and new lead compounds and scaffolds from TCM. Combined chemical and biological studies on the effective TCM have provided unprecedented opportunities for the development of new drugs and the modernisation of TCM. In such circumstances, many in vitro and in vivo studies have been conducted to explore the therapeutic effect of the natural remedies in HA. However, the therapeutic efficacy of these natural remedies has yet to be demonstrated or translated into clinical practice. Hence, this review aims to synthesise the evidence in pre-clinical and clinical research on natural remedies published from June 2007 to July 2020 to examine if these experimental studies may provide a mechanistic framework for developing treatments for HA towards clinical trials.

## Methods

### Search strategy

We conducted a systematic search of Web of Science, PubMed and Science Direct Scopus electronic databases for relevant articles published from June 2007 to July 2020. Google Scholar was used to identify any articles not included in the above databases. The search terms included a combination of the following keywords: algae, alternative medicine, ataxia, basidiomycete, complementary medicine, herbal, herbs, medicinal plants, medicinal mushrooms, mushrooms, natural products, plants and seaweed. The structured search strategy was designed to include all papers that evaluated the use of natural remedies as a treatment for HA published from June 2007 to July 2020.

### Eligibility criteria

Studies were considered if they met the following inclusion criteria: (1) pre-clinical (in vitro and in vivo studies) and clinical studies, (2) study model of HA as the primary disorder, (3) full-text available and (4) articles published in English. The exclusion criteria included: (1) studies with ataxia as the secondary symptom, (2) studies targeting non-motor symptoms, (3) studies using chemicals to induce ataxia, (4) review articles, (5) conference abstracts or proceedings, (6) full-text not available and (7) articles published in another language other than English.

### Data extraction and analysis

Collection and extraction of data were conducted according to the Preferred Reporting Items for Systematic Review and Meta-Analyses (PRISMA). Two researchers (M.W.L.P and S.Y.L) searched and independently reviewed all the relevant studies and selected the ones meeting the inclusion criteria. Disagreements on the eligibility or on the extraction of data were resolved through discussions between the two researchers. The other team members made the final decision if the two researchers could not reach an agreement. Due to inconsistencies in the methodology or experimental design in the retrieved studies, findings were extracted independently and narrated to the best of our ability.

## Results

### Study selection

**Fig. 1:**
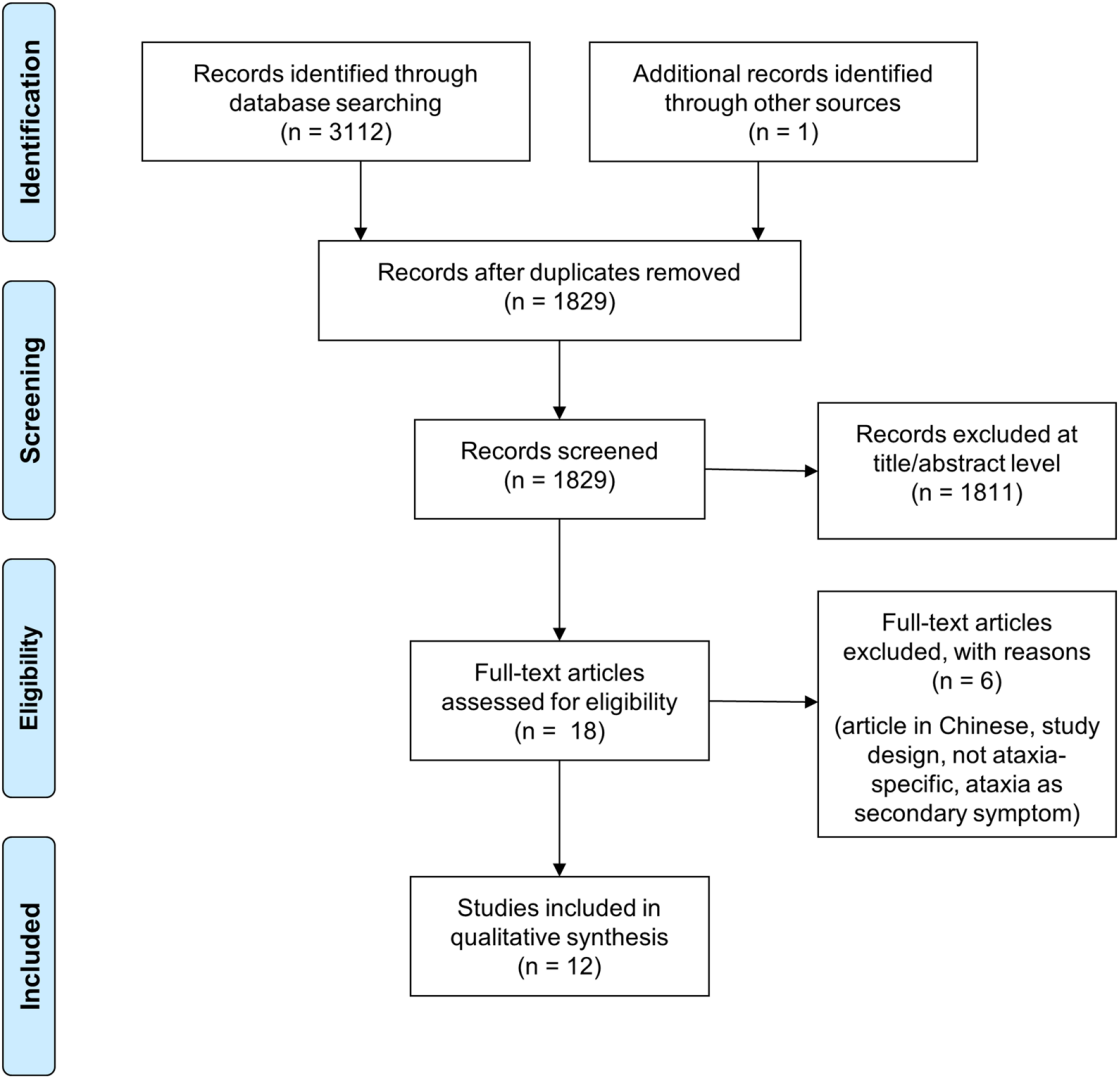
PRISMA flowchart for identification of relevant studies

The systematic literature search yielded 3113 results from the three databases. After eliminating duplicate studies, 1829 studies remained and were further evaluated by screening the titles and abstracts. The full-texts of 18 selected studies were retrieved for further assessment and evaluation. Six articles were excluded at this stage for various reasons including language, study design, not ataxia-specific, or ataxia was a secondary symptom. Overall, 12 studies (ten pre-clinical studies and two clinical studies) were eligible for inclusion in this systematic review. Figure 1 shows the PRISMA flow chart for the identification of relevant studies.

**Fig. 2:**
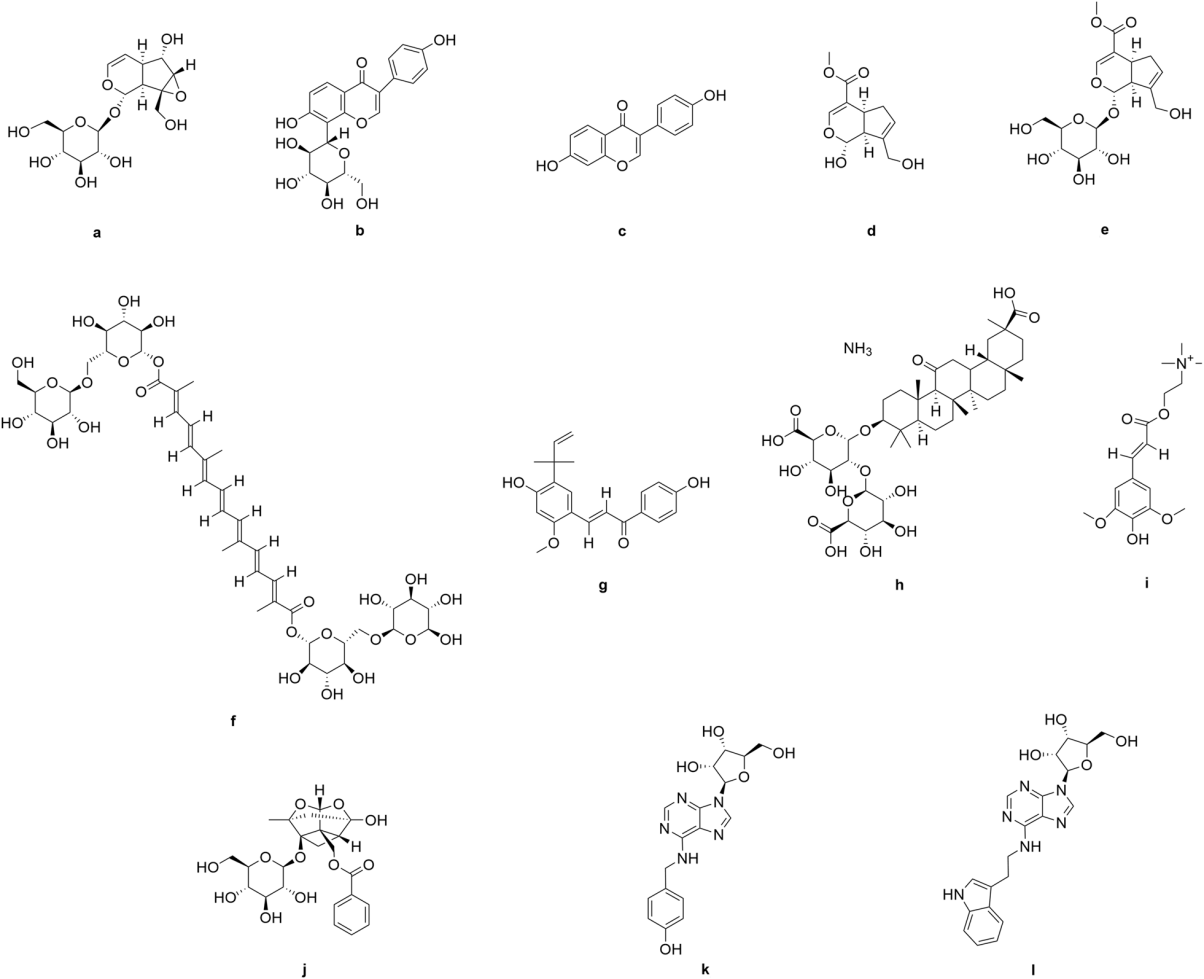
Chemical structures of the identified compounds with bioactive effects against HA

Based on the retrieved studies, we found several natural remedies, including medicinal plants, herbal formulations and medicinal mushrooms, had been investigated as treatments in only two subtypes of HA, SCA and FRDA. The list of natural remedies and their bioactive effects against HA are summarised in Table 2. The chemical constituents of the identified natural remedies are shown in Fig. 2; namely the catalpol (Fig. 2a) from *Rehmannia glutinosa*; puerarin (Fig. 2b) and daidzein (Fig. 2c) from *Pueraria lobata*; genipin (Fig. 2d), geniposide (Fig. 2e) and crocin (Fig. 2f) from *Gardenia jasminoides*; licochalcone A (Fig. 2g) and ammonium glycyrrhizinate (Fig. 2h) from *Glycyrrhiza inflata*; sinapine (Fig. 2i) in rapeseed pomace; and paeoniflorin (Fig. 2j) from *Paeonia lactiflora*; and T1-11 (N^6^-(4-Hydroxybenzyl) adenosine) (Fig. 2k) and JMF-1907 (N^6^-(3-Indolylethyl) adenosine) (Fig. 2l) from *Gastrodia elata.*

**Table 2:** Natural remedies and their therapeutic effects

Disorder	Natural Remedies	Compounds	Model	Finding	Mode of Action	Reference
Medicinal plant						
SCA3	*Rehmannia glutinosa*	Catalpol	HEK-293 and SH-SY5Y cells	Promoted proteasome activity, suppressed polyQ aggregation, prevented cell death	UPS	[55]
	*Pueraria lobate*	Puerarin and daidzein	iPSCs	Promoted proteasome activity, suppressed polyQ aggregation, prevented cell death		[56]
	*Gastrodia elata*	T1-11 and JMF-1907	Mice	Promoted proteasome activity, prevented cell death, restored motor function		[57]
	*Gardenia jasminoides*	Genipin, geniposide and crocin	HEK-293 and SH-SY5Y cells	Promoted the expression of antioxidant genes, suppressed ROS level	NRF2 signalling pathway	[58]
	*Glycyrrhiza inflata*	Licochalcone A and AMGZ		Promoted the expression of antioxidant genes, suppressed ROS level, suppressed polyQ aggregation		[59]
	*Paeonia lactiflora*	Paeoniflorin		Promoted expression of heat shock proteins, suppressed polyQ aggregation, restored motor function	N/A	[60]
	*Brassica napus*	Sinapine	Roundworm	Promoted expression of antioxidant genes, suppressed ROS level, restored motor function	N/A	[61]
SCA17	*Ginkgo biloba*	N/A	SH-SY5Y cells and mice	Prevented calcium influx, prevented cell death	Calcium homeostasis	[62]
Herbal formulation						
SCA6	Zhengan Xifeng Tang	N/A	Human	Restored motor function	N/A	[63, 64]
SCA17	Shaoyao Gancao Tang	N/A	HEK-293 and SH-SY5Y cells	Promoted expression of antioxidant genes, prevented cell death, restored mitochondrial biogenesis, restored motor function	N/A	[65]
Medicinal mushroom						
FRDA	*Hericium erinaceus*	N/A	Fibroblast	Prevented antioxidant depletion, prevented cell death	N/A	[66]

*SCA* spinocerebellar ataxia, *HEK-293* human embryonic kidney 293 cells, *UPS* ubiquitin–proteasome system, *iPSCs* induced-pluripotent stem cells, T1-11, N^6^-(4-Hydroxybenzyl) adenosine; JMF-1907, N^6^-(3-Indolylethyl) adenosine; *NRF2* nuclear factor erythroid 2–related factor 2; *AMGZ* ammonium glycyrrhizinate; *FRDA* Friedreich’s ataxia; *N/A* not applicable

## Discussion

### Mode of action

#### Activation of ubiquitin–proteasome system (UPS)

In SCA, abnormal expansion of CAG repeats in respective coding regions of SCA leads to elongated translation of the polyglutamine (polyQ) tract. When the expansion reaches a specific threshold, it causes the abnormal configuration of the encoded polyQ protein, leading to protein aggregation and nuclear inclusions, a pathological hallmark of SCA [67]. The presence of polyQ aggregates are detrimental to cellular processes, and contributes to cellular dysfunction and death [68, 69]. The UPS plays a pivotal role in intracellular protein homeostasis by clearing misfolded and defective proteins [69]. Furthermore, UPS is one of the major regulators of cell cycle progression and apoptosis [70]. Evidence shows polyQ proteins are susceptible to ubiquitination and proteasome degradation [71–74]. However, it was shown that the accumulation of polyQ aggregates compromised the function of UPS in SCA [55–57] potentially through deubiquitylation [66], leading to further accumulation of aggregates that would otherwise have been removed. Therefore, restoration of UPS function using natural remedies may serve as a therapeutic strategy against polyQ-induced SCA.

In a study by Chen et al. [55], the anti-aggregation effects of catalpol derived from *Rehmannia glutinosa*, and daidzein and puerarin derived from *Pueraria lobata* were investigated in in vitro models of SCA3. Carbobenzoxy-L-leucyl-L-leucyl-L-leucinal (MG132), a proteasome inhibitor, was used to treat human embryonic kidney 293 cells (HEK-293) and neuroblastoma SH-SY5Y cells expressing mutant ataxin-3 proteins. Pre-treatment with 0.1 μM catalpol, puerarin or daidzein for 24 h showed no cytotoxicity against the cells, as indicated by 3-(4,5-dimethylthiazol-2-yl)-2,5-diphenyl tetrazolium bromide (MTT) assay. The compounds were able to restore UPS function and effectively enhance proteasome activity in the cells, as evidenced by low green fluorescent protein (GFP) intensity. Additionally, the compounds suppressed polyQ aggregation induced by mutant ataxin-3 protein, as detected by Western blot analysis. Following pre-treatment with the test compounds, no autophagy activity was observed in the cells, as evidenced by an insignificant increase in red fluorescent protein (DsRed-LC3) intensity, suggesting the anti-aggregation ability of the compounds were due to UPS activation. Furthermore, the compounds also suppressed the expression of pro-apoptotic marker BCL-2-associated X (BAX) protein and promoted the expression of anti-apoptotic marker B-cell lymphoma 2 (BCL2) protein. Caspase-3 activity was also reduced, which prevented polyQ-induced cell death [55]. These findings were in line with another study by Chen et al. [56] using an in vitro model of SCA3 with MG132-induced pluripotent stem cells (iPSCs). Pre-treatment with 50 μM daidzein for 24 h restored proteasome activity in the cells. Additionally, daidzein was able to suppress MG132-induced cell death, as indicated by lactate dehydrogenase (LDH) assay [56].

A study by Chou et al. [57] investigated the proteasome enhancing ability of T1-11 (N^6^-(4-Hydroxybenzyl) adenosine), a compound derived from *Gastrodia elata*, and JMF-1907 (N^6^-(3-Indolylethyl) adenosine), a synthetic analogue of the former. They used an in vivo model of SCA3 in transgenic mice expressing mutant ataxin-3 protein. Oral administration of 50 mg/kg T1-11 or JMF-1907 for 3 months prevented neuronal cell death in the pontine nuclei by reversing the induced expression of BAX protein and decreased expression of B-cell lymphoma-extra large (BCL-xL) protein. Additionally, proteasome activity was significantly enhanced in the cerebellum and pontine nuclei in the treatment group, as indicated by increased levels of 7-amino-4-methylcoumarin (AMC). An improvement in motor functions was also observed in the treatment group compared to untreated animals, as indicated by the rotarod test [57].

**Fig. 3:**
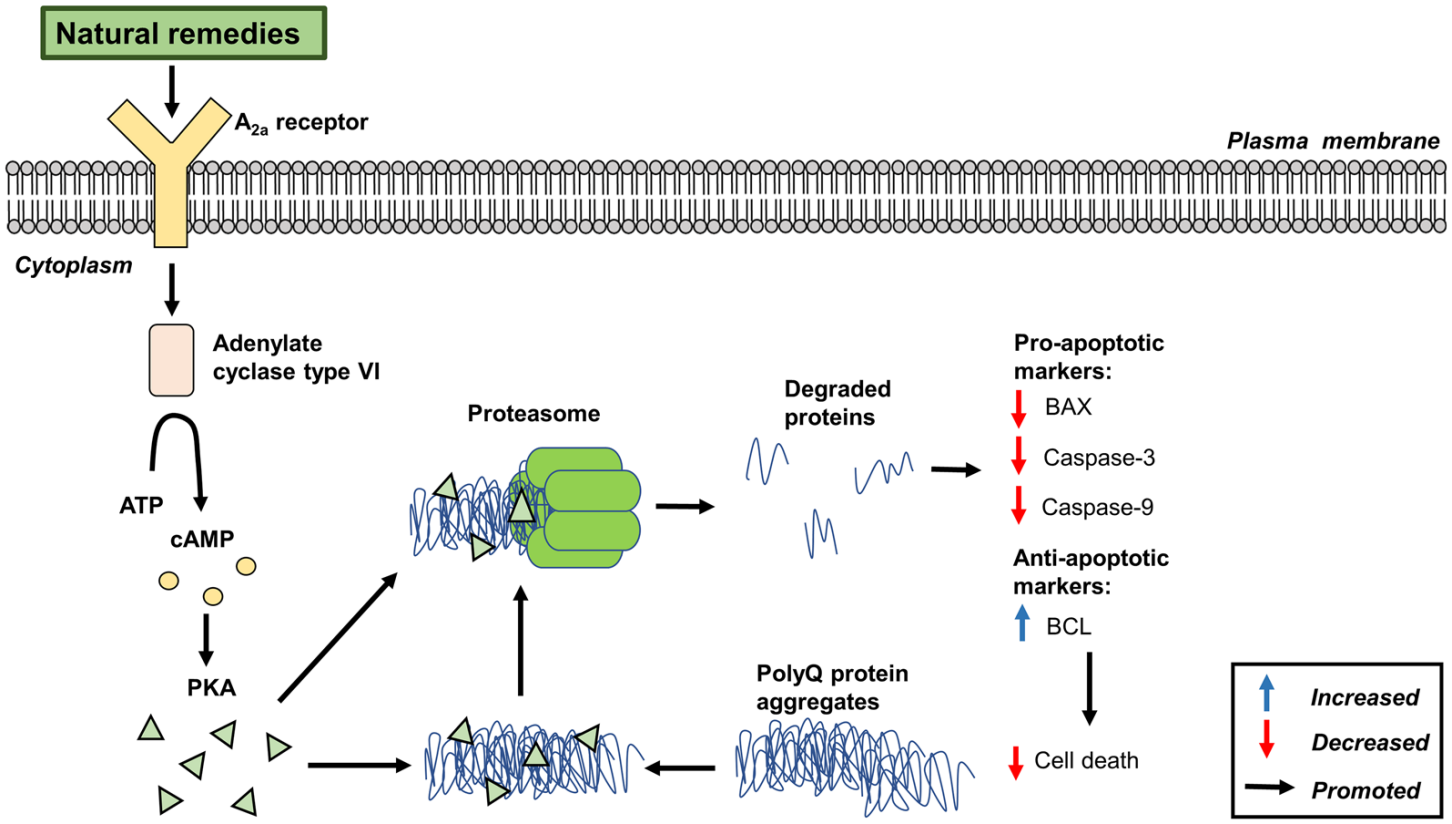
Schematic diagram of the activation of UPS by natural remedies against polyQ-induced SCA. *ATP* adenosine triphosphate, c*AMP* cyclic adenosine monophosphate, PKA protein kinase, *PolyQ* polyglutamine, *BAX* BCL-2-associated X, *BCL* B-cell lymphoma

A schematic of the possible activation of the UPS signalling pathway by natural remedies is shown in Fig. 3. Natural remedies have been shown to activate UPS, resulting in the suppression of polyQ aggregation and motor dysfunction associated with SCA [55–57]. The cAMP/PKA pathway is known to activate UPS and promote proteasome activity [75] involving the activation of adenylate cyclase 6 through the A_2A_ receptor [76]. Chou et al. [57] reported that the activation of A_2A_ receptor by T1-11 and JMF-1907 could enhance proteasome activity and promote degradation of ubiquitin proteins. Similar findings were later reported by Chen et al. [55, 56] using puerarin and daidzein compounds extracted from *R. glutinosa* and *P. lobata*. Inhibition of A_2A_ receptor using an A_2A_ antagonist blocked the effects induced by T1-11 and JMF-1907. This suggests the stimulation of A_2A_ receptor is required for UPS activation [57]. The suppression of polyQ aggregation was reported to prevent polyQ-induced cell death via suppression of BCL-2-associated X (BAX), and caspase-3 and -9 activities, while enhancing B-cell lymphoma 2 (BCL) expression [55–57].

#### Activation of antioxidant pathways

Oxidative damage occurs when reactive oxygen species (ROS) generation exceeds the antioxidant capacity of intracellular antioxidant enzymes [77]. It was shown that the accumulation of polyQ aggregates can cause the depletion of antioxidants including glutathione (GSH), superoxide dismutase (SOD) and catalase (CAT), contributing to the accumulation of ROS and oxidative damage [78, 79]. Patients diagnosed with SCA3 were reported to have reduced levels of glutathione and other thiols, which increased their susceptibility to oxidative damage [16, 80]. This condition may contribute to ROS-induced DNA damage and cell death, leading to motor dysfunctions associated with the disorder [59, 81, 82] Furthermore, the presence of ROS promoted protein aggregation, causing further generation of ROS [59]. Therefore, restoration of endogenous antioxidant levels by natural remedies could serve as a therapeutic strategy against SCAs.

Three derived compounds genipin, geniposide and crocin and the aqueous extract of *Gardenia jasminoides* were investigated for their antioxidant activities in in vitro models of SCA3 using HEK-293 and SH-SY5Y cells expressing mutant ataxin-3 proteins [58]. The presence of polyQ aggregates in the cells were observed to suppress the expression of nuclear factor erythroid 2-related factor 2 (NRF2), an antioxidant marker. Pre-treatment with aqueous extract (1-10 g/mL), genipin (50–500 nM), geniposide (500 nM) or crocin (100 nM) for 8 h showed no cytotoxicity in the cells, as indicated by MTT assay. Pre-treatment with the extract or compounds restored the depleted expression of NRF2 and downstream antioxidant enzymes NAD(P)H quinone dehydrogenase 1 (NQO1), glutamate-cysteine ligase catalytic subunit (GCLC), and glutathione S-transferase pi 1 (GSTP1), while suppressing ROS levels and polyQ aggregation, as detected by low ROS fluorescence and GFP intensities. Overexpression of NRF2 in cells suppressed polyQ aggregation, whereas knockdown of its gene promoted polyQ aggregation. These findings suggest the involvement of antioxidant genes in the anti-aggregation effects in cells. Pre-treatment with extract or compounds did not significantly change DPPH (2,2-diphenyl-1-picrylhydrazyl) scavenging activity, suggesting DPPH radical scavenging is not a major factor in the anti-aggregation effects [58].

Similar findings were reported by Chen et al. [59] using the aqueous extract of *Glycyrrhiza inflata* and its compounds licochalcone A and ammonium glycyrrhizinate (AMGZ) in in vitro models of SCA3 using HEK-293 and SH-SY5Y cells expressing mutant ataxin-3 proteins. Pre-treatment with aqueous extract (50 µg/mL), licochalcone A (10 nM) or AMGZ (1 µM) for 8 h showed low cytotoxicity in cells, as indicated by MTT assay. However, the extract or compounds upregulated the expression of peroxisome proliferator-activated receptor γ coactivator 1α (PGC-1α), a regulator of the mitochondrial antioxidant defence system, and its downstream targets, NRF2, NQO1, SOD2, cytochrome c (CYCS) and heme oxygenase 1 (HMOX1), as detected by Western blot analysis. Oxidative damage was reduced through promotion of the glutathione/oxidised glutathione (GSH/GSSG) ratio, suppression of ROS levels and polyQ aggregation [59].

Pohl et al. [61] investigated the antioxidant activities of ethanol extract from rapeseed pomace, a by-product of *Brassica napus* using Soxhlet extraction. Administration of ethanol extract (1-5 mg/mL) in an in vivo model of SCA3 using transgenic *Caenorhabditis elegans* expressing mutant ataxin-3 protein showed no toxicity, as detected by food clearance assay. Ethanol extract treatment promoted the expression of glutathione S-transferase 4 (GST4) and SOD3, which are involved in GSH metabolism and mitochondrial manganese SOD synthesis, respectively. Furthermore, ethanol extract improved motor function in transgenic *C. elegans* comparable with the wild type, as observed by a motility assay. Knockdown of GST4 gene in *C. elegans* abolished the improvement in motor function by ethanol extract. Pohl et al. [61] suggested that the positive effects of rapeseed pomace extract were dependent on GST4 expression. Moreover, sinapine, a major compound derived from the ethanol extract, also demonstrated improvement in motor function of transgenic *C. elegans*, but it was less effective than the whole extract. This indicates additional compounds in conjunction with sinapine contributed to the positive effects of ethanol extract [61].

Shaoyao Gancao Tang is a Chinese traditional herbal formulation consisting of *Paeonia lactiflora* and *Glycyrrhiza uralensis* in a 1:1 ratio. In a study by Chen et al. [65], the antioxidant activities of the formulation were evaluated in in vitro models of SCA17 using HEK 293 and SH-SY5Y cells expressing mutant TATA-box binding protein (TBP), and in vivo transgenic mouse model of SCA17 overexpressing TBP. Pre-treatment with the formulation (0.001–100 µg/mL) in cells for 8 h demonstrated no cytotoxicity, as indicated by MTT assay. The formulation was able to restore the expression of PGC-1α and NRF2, and their downstream targets, CYCS, GCLC and NQO1, as detected by Western blot analysis. Furthermore, suppression of polyQ aggregation was observed in cells, as shown by low GFP intensity. Administration of 0.4% Shaoyao Gancao Tang in the drinking water of the mouse model improved gait coordination and suppressed hyperactivity, as indicated by footprint and locomotor test, respectively [65].

Zhengan Xifeng Tang is a Chinese traditional herbal formulation consisting of various mixtures of medicinal plants. The positive effects of the formulation were investigated by Okabe et al. [63] in a 60-year-old female patient diagnosed with typical SCA6. Oral administration of the formulation for 2 months showed visible alleviation of ataxia symptoms including vertigo and titubation. Motor function was also observed to be improved, as indicated by the International Cooperative Ataxia Rating Scale (ICARS). At 1 year after treatment discontinuation, the patient experienced a relapse in ataxia symptoms, although re-administration of the formulation alleviated the deteriorating symptoms. However, the patient relapsed a second time after another 15 months after treatment discontinuation, which was again alleviated by the treatment [63]. Similar findings were demonstrated in another study by Okabe [64] in 71-year-old female patient diagnosed with typical SCA6. Although the mechanisms of Zhengan Xifeng Tang on SCA6 were not identified from these studies, it was suggested that the positive effects may be attributed to the antioxidant and anticonvulsant activity of the herb formulation [63, 64].

**Fig. 4:**
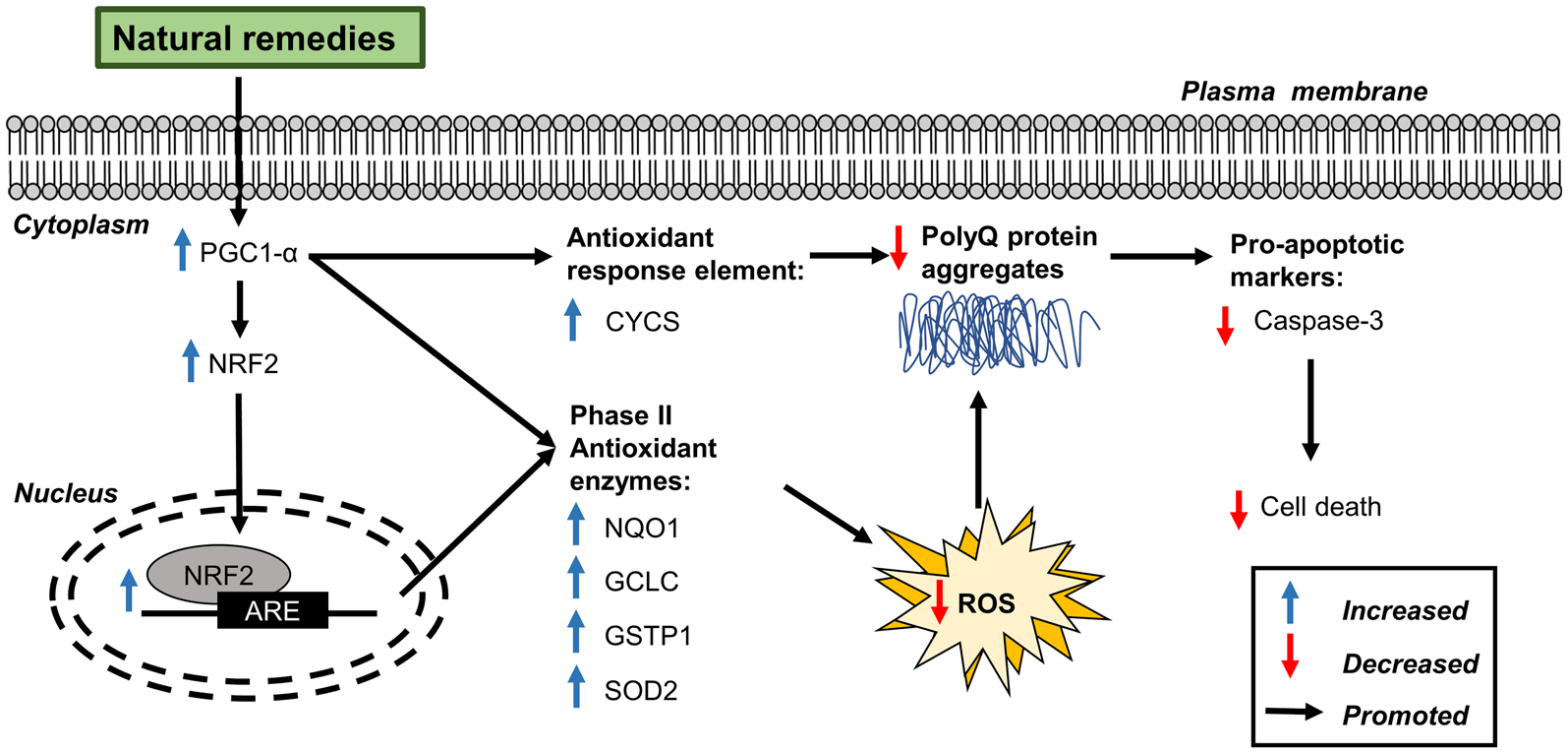
Schematic diagram of the activation of the antioxidant signalling pathway by natural remedies against polyQ-induced SCA. *PGC-1α* peroxisome proliferator-activated receptor γ, *NRF2* nuclear factor erythroid 2-related factor 2, *ARE* antioxidant response element, *CYCS* cytochrome c, *NQO1* NAD(P)H Quinone Dehydrogenase 1, *GCLC* glutamate-cysteine ligase catalytic subunit, *GSTP1* glutathione S-transferase pi 1, *SOD2* superoxide dismutase 2, *PolyQ* polyglutamine, *ROS* reactive oxygen species

The activation of the antioxidant pathway by natural remedies is shown in Fig. 4. The antioxidant activities of natural remedies were shown to have therapeutic effects against cell damage in SCA through the activation of NRF2/ARE (antioxidant response element). This antioxidant pathway involved the upregulation of various phase II detoxifying/antioxidant enzymes that attenuated the oxidative damage and prevented ROS-induced cell death [83]. In a study by Chen et al. [65], Shaoyao Gancao Tang upregulated the expression of NRF2 and PGC-1α, a coactivator of NRF2, which suppressed ROS levels in vitro and restored motor function in transgenic SCA17 mice. Studies by Chang et al. [58] and Chen et al. [59] showed that *G. jasminoides* and *G. inflata* could activate downstream targets of NRF2 and PGC-1α (e.g., SOD2, CYCS, NAD(P)H, NQO1, GCLC and GSTP1), which effectively suppressed ROS levels and polyQ aggregation. In contrast, knockdown of PGC-1α and/or NRF2 promoted aggregation and attenuated the anti-aggregation activity of Shaoyao Gancao Tang, *G. jasminoides* and *G. inflata*, which suggest PGC-1α and NRF2 are involved in the positive effects on SCA [58, 59, 65].

Friedreich’s ataxia (FRDA) is an autosomal recessive disorder caused by the abnormal expansion of GAA repeats contributing to suppressed expression of frataxin (FXN) protein [84]. Evidence shows that frataxin deficiency in FRDA impairs the intracellular antioxidant defence system, resulting in decreased GSH levels [85–87] and expressions of antioxidant enzymes including SOD, CAT, glutaredoxin, and thioredoxin [88]. Additionally, excessive ROS is generated by an iron-dependent Fenton reaction and mitochondrial dysfunction. The compromised antioxidant defence system and ROS accumulation contribute to the unregulated increase in oxidative damage, leading to DNA damage and cell death [89–92].

Lew et al. [66] investigated the antioxidant activities of a standardised aqueous extract of *Hericium erinaceus* in an in vitro model of FRDA involving L-Buthionine sulfoximine (L-BSO)-induced human dermal fibroblast expressing abnormal expansion of GAA triplet repeat. L-Buthionine sulfoximine is an inhibitor of γ-glutamylcysteine synthetase, which plays a role in GSH biogenesis. Cells treated with L-BSO had reduced levels of GSH, which increased the susceptibility of cells to oxidative damage [93]. Treatment of cells with the extract (32 mg/mL) for 24 h restored the GSH depletion, as shown by an increased GSH/GSSG ratio. The extract also reduced the lactate dehydrogenase (LDH) level and apoptotic body formation, as detected by LDH assay and Hoechst 33258 staining, respectively. The findings suggest the extract was able to prevent oxidative damage-induced cell death by preventing GSH depletion [66].

#### Maintenance of intracellular calcium homeostasis

Calcium ions are important signalling molecules that control a wide variety of cellular processes including muscle control, cell signalling and cell growth [94]. Accumulating evidence shows that polyQ aggregates impair calcium homeostasis and induce abnormal calcium ion influx in Purkinje cells through ionotropic α-amino-3-hydroxyl-5-methyl-4-isoxazole-propionate (AMPA) receptors and metabotropic glutamate receptors (mGluR) [95–98]. The accumulation of calcium ions leads to the formation of microaggregates, eventually forming larger inclusion bodies, leading to neuronal degeneration and cell death, and compromised cerebellar output and motor function [67]. Natural remedies that can restore calcium homeostasis could potentially treat polyQ disorders.

**Fig. 5:**
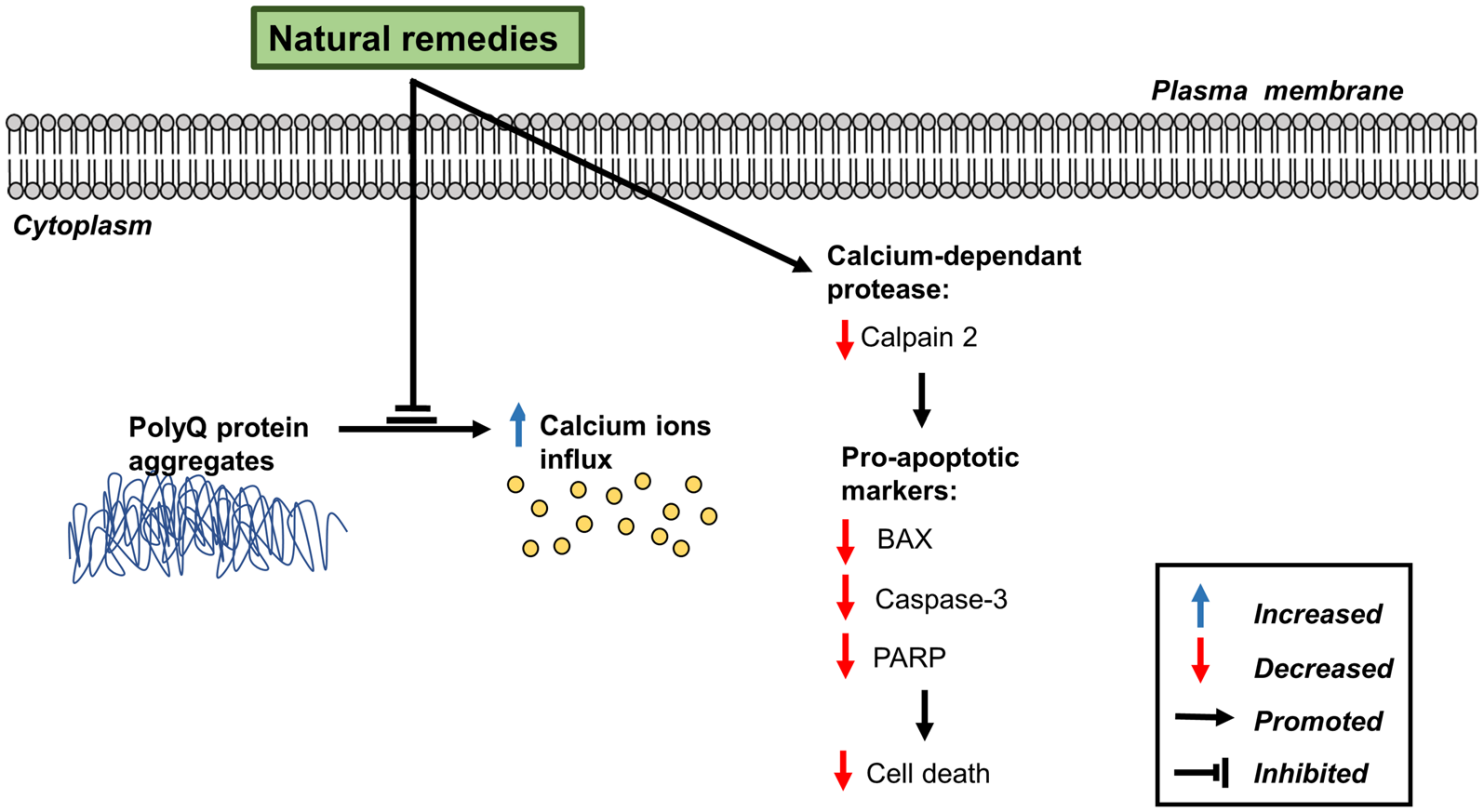
Schematic diagram of the maintenance of intracellular calcium homeostasis by natural remedies against polyQ-induced SCA. *BAX* BCL-2 associated X, *PARP* poly (adenosine diphosphate-ribose) polymerase

A study by Huang et al. [62] investigated the positive effects of EGb 761, a standardised extract of *Ginkgo biloba*, in in vitro models of SCA17 using HEK 293 and SH-SY5Y cells expressing mutant TATA-box binding protein (TBP), and an in vivo transgenic mouse model overexpressing TBP. Pre-treatment with EGb 761 (20 μg/mL) for 1 h restored cellular calcium homeostasis and suppressed calcium influx, as indicated by reduced calcium green fluorescence intensity. Additionally, there was a reduction in calcium-dependent pro-apoptotic marker expressions, including calpain 2, BAX, caspase 3 and poly (adenosine diphosphate-ribose) polymerase (PARP), as detected by Western blot analysis. Intraperitoneal injection of EGb 761 (100 mg/kg) in transgenic mice demonstrated improved motor function by rotarod test. Moreover, EGb 761 prevented the disruption of calbindin integrity, as indicated by immunohistochemistry analysis [62]. Calbindins are major proteins expressed on neurons such as Purkinje cells, which play a role in motor function, calcium homeostasis and cell survival [99, 100]. Similar findings were also reported by Chen et al. [65] using Shaoyao Gancao Tang in Purkinje cells of SCA17 mice detected by calbindin staining. A schematic of the maintenance of intracellular calcium homeostasis by natural remedies is shown in Fig. 5.

#### Regulation of chaperones

Heat shock proteins (HSPs) are evolutionarily conserved chaperones ubiquitous in organisms and are responsible for cell survival by facilitating protein assembly, folding, translocation, stabilisation and degradation [101, 102]. Previous studies by Chen et al. [103] and Lee et al. [104] demonstrated the presence of TBP induced the sequestration of nuclear transcription factor Y (NFY) complexes and suppressed its downstream targets including the heat shock 70 kDa protein (HSPA) 5 and 8. Studies also showed that upregulation of HSPs could suppress polyQ aggregation and protect against polyQ-induced toxicity [105–109]. Natural remedies that can restore HSP expression and function could serve as an alternative therapeutic option for polyQ-induced SCAs.

**Fig. 6:**
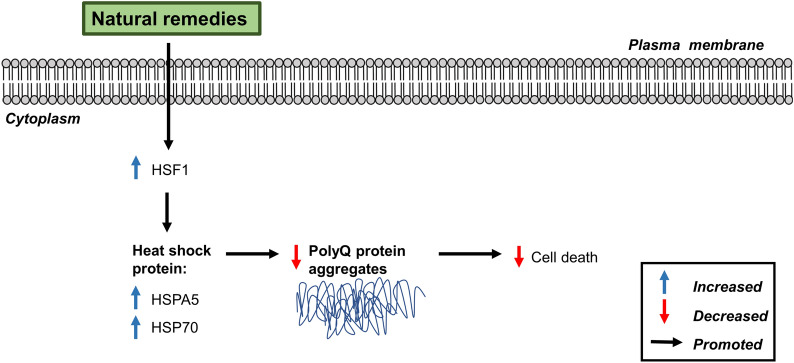
Schematic diagram of the regulation of chaperones by natural remedies against polyQ-induced SCA. *HSF1* heat shock transcription factor 1, *HSPA5* heat shock 70 kDa protein 5, *HSP70* heat shock protein 70

Chang et al. [60] investigated the effects of *Paeonia lactiflora* and its major compound paeoniflorin on HSPs in in vitro models of SCA3 using HEK-293 and SH-SY5Y cells expressing mutant ataxin-3 proteins. Pre-treatment with *P. lactiflora* (2-50 *μ*g/mL) for 24 h showed no cytotoxicity, as assessed by MTT assay. The extract and compound upregulated the expression of heat shock transcription factor 1 (HSF1) and heat shock protein 70 (HSP70) chaperone in cells. Additionally, suppression of polyQ aggregation was observed in the cells, as shown by low GFP intensity. Overexpression of HSP1 also resulted in suppressed polyQ aggregation, suggesting that the positive effects of the extract and compound were modulated by HSPs. The role of HSPs in suppressing polyQ aggregation was further supported by Chen et al. [65] in an in vivo model of SCA17. Administration of Shaoyao Gancao Tang promoted HSPA5 expression and suppressed TBP protein aggregation in transgenic mice leading to improved motor function in the rotarod test [65]. The regulation of chaperones by natural remedies is shown in Fig. 6.

## Limitations and future prospects

There are valid concerns on the use of natural remedies in HA drug discovery and development. Natural remedies are often initially experimented as crude extracts and due to the high abundance of non-desirable components, bioassay-guided isolation of target compounds must be performed with subsequent identification [110, 111]. Furthermore, metal impurities, fluorescence-interfering, the presence of non-polar or polar compounds in the extracts may provide inaccurate results for some bioassays. Therefore, such compounds must first be removed depending on the assay system to avoid interference [112]. The process requires more time and effort as compared to high-throughput screening of chemical compound libraries [110, 111].

Another impediment in its acceptance is the lack of standard profile for quality control, cost per sample, complexity of resupply and variation of chemical composition. Natural remedies harvested from the wild are susceptible to environmental factors such as deforestation, pollution, global warming and anthropic pressure which hinders their habitat and limits availability. In addition, discrepancy in growth requirements such as soil composition, weather, altitude and storage conditions contribute to variation of chemical composition. The composition may also be affected during processing and isolation as some compounds may undergo transformation and degradation [110–112]. The ecological issues can be resolved through the generation of a controlled cultivation system which provides optimal yield and uniform composition, preventing batch to batch variation [113]. Nevertheless, majority of the natural remedies worldwide are still harvested from the wild [112]. This is due to the high commercial risk associated with generating the system as the market is unpredictable and the duration required for the establishment is long [113].

It is worthy to emphasise that HA is a rare genetic disorder with severe survival outcomes; hence, development of translational models of human-specific HA is rather limited. Studies of innovative treatment with small sample size by individual laboratories should by no means be substituted by multi-centre pre-clinical mouse studies. Nevertheless, the limitations of such smaller studies should be clearly defined by differentiating between proof-of-concept studies and pre-clinical testing studies.

Although various forms of HA have been identified, only four subtypes of HA (SCA3, 6, 17 and FRDA) were investigated with natural remedies in the reviewed studies. Furthermore, the studies demonstrating the positive effects of natural remedies on HA were largely conducted with pre-clinical models such as in vitro cell culture models and in vivo animal models. While these models have facilitated the understanding of mechanisms and signalling pathways in the treatment of HA, the results need to be validated in human studies. Additionally, although there are no models that can reproduce all symptoms of HA patients or cellular mechanisms involved, mammals (*Drosophila*, transgenic and knockout mice) and mammalian cell lines (patient-derived cells, iPSCs and iPSC-derived cells) are currently the most useful models, owing to their close physiological features to HA diseases [114, 115]. Due to the complexity and heterogeneity of HA pathogenesis, current approaches are focusing on the development of therapeutic strategies that target different stages of the cellular pathogenesis, thus making comparison across the studies challenging [115]. Moreover, it is difficult to compare transgenic mice derived from different mouse strains as the manifestation of the targeted mutations may be interfered by their phenotypic traits of the original strain [116].

In spite of the fact that natural remedies are shown to produce significant improvement in HA patients, the results of such studies are limited by the sample size and possible bias to ethnicity and gender. Clinical studies by Okabe et al. [63] and Okabe [64] were each conducted with only one test subject. Furthermore, both test subjects were elderly Japanese female aged 60 and 71. The limited number of test subject and lack of diversity in terms of age and ethnicity may affect the reliability of the findings [117]. Another major limitation is the lack of safety and efficacy profile for the natural remedies and compounds studied in this review. It is not known whether the dosage of decoctions with complex formulations and compounds administered may lead to potential toxicity, adverse effects or will be present at the intended site of action in sufficient concentration [118–120]. This is also further complicated with the lack of understanding of the molecular targets and mechanism involving the natural remedies [120]. Greater understanding of the pharmacokinetic and pharmacodynamics profiles of these compounds will support more effective clinical trials in the future.

A more systematic approach in cataloguing the potential effects of natural remedies/compounds will speed up the drug discovery process, in order to have more investigations done on unexplored compounds. Detailed mode of actions for each compound can be delineated using high throughput methods such as NGS coupled with various other confirmative studies such as transcriptomic validation, protein translation and function as well as epigenetic studies. Integrative analysis of such ‘omics’ data will allow researchers with an unprecedented speed in understanding the underlying pathophysiology this rare disease, developing effective biomarkers and pursuing meaningful clinical testing [121]. Studies on specific HA can be undertaken using relevant genetic resources or repositories of biologic samples. NGS can be implemented on HA research in identifying and validating potential targets through epigenetic studies, differential gene expression, genome sequencing and protein translation rates. Moreover, NGS sequencing can be used to discover biomarkers that can reflect the mechanism of action regulated by the natural remedy or compound [122]. These pre-clinical studies can provide proof of concept and mechanism in establishing the pharmacodynamic parameters of these remedies and compounds prior to clinical testing.

As pre-clinical and clinical studies are currently lacking, effort should be directed towards testing using polyQ animal models and randomised controlled trials, respectively, including for forms of HA not yet studied. Improved studies should be done where study conclusions are uncertain due to methodological insufficiencies or discrepancies. Comparative studies should be done with current treatments versus natural remedies (single compound or the mixture) to ascertain the potential of the latter as supplements or alternative treatments for HA. Additionally, further studies can be conducted towards investigating whether the natural remedies could modulate HA through other pathways not discussed in this review.

In addition, a successful planning and execution of a research consortium involving Friedreich’s Ataxia Research Alliance (FARA) and National Ataxia Foundation (NAF) opens new possibilities for HA research. Such effort will foster cross-nationals translational research in the development of alternative treatments for HA. Multidisciplinary team involvement and input form the allied health professionals are also critical in the management of HA, particularly in the palliative stage. Indeed, all these efforts will require strong commitment not only from the researchers, but also the patients diagnosed with HA as well as their family members.

## Conclusion

In conclusion, the systematic review provides an overview of the available evidence from pre-clinical and clinical trials pertaining to the natural remedies in improving or halting the progression of HA. Nevertheless, there has been an increasing concern by the clinicians about the potential of interactions of natural remedies with prescribed drugs. The mechanisms underlying natural remedies-drug interactions are always difficult to predict. Another issue is the general lack of understanding on the composition and pharmacological actions of natural remedies. Therefore, research evidence is necessary to ensure its safety and effectiveness and to warrant the integration of natural remedies into evidence-based clinical practice.

## Data Availability

Not applicable.

## Abbreviations

HA: Hereditary ataxia
SCA: Spinocerebellar ataxia
CAG: Cytosine-Adenine-Guanine
EA: Episodic ataxia
FRDA: Friedreich’s ataxia
AT: Ataxia-telangiectasia
AOA: Ataxia oculomotor apraxia
FXTAS: Fragile X tremor-ataxia syndrome
TCM: Traditional Chinese medicine
PRISMA: Preferred Reporting Items for Systematic Review and Meta-Analyses
HEK-293: Human embryonic kidney 293 cells
PolyQ: Polyglutamine
UPS: Ubiquitin-proteasome system
cAMP: Cyclic adenosine monophosphate
PKA: Protein kinase
ROS: Reactive oxygen species
NRF2: Nuclear factor erythroid 2–related factor 2
AMGZ: Ammonium glycyrrhizinate
MTT: 3-(4,5-dimethylthiazol-2-yl)-2,5-diphenyl tetrazolium bromide
GFP: Green fluorescent protein
LDH: Lactate dehydrogenase
BAX: BCL-2-associated X protein
BCL2: B cell lymphoma 2
T1-11: N^6^-(4-Hydroxybenzyl) adenosine
JMF1907: N^6^-(3-Indolylethyl) adenosine
GSH: Glutathione
SOD: Superoxide dismutase
CAT: Catalase
NQO1: NAD(P)H quinone dehydrogenase 1
GCLC: Glutamate-cysteine ligase catalytic subunit
GSTP1: Glutathione S-transferase pi 1
CYCS: Cytochrome c
GSSG: Oxidised glutathione
GST4: Glutathione S-transferase 4
TBP: TATA-box binding protein
PGC-1α: Peroxisome proliferator-activated receptor γ coactivator 1α
HSP: Heat shock protein
